# Association between biological rhythm disruption and suicidal ideation in patients with major depressive disorder: a chain mediation of neuroticism and emotion regulation difficulties

**DOI:** 10.3389/fpsyt.2026.1895657

**Published:** 2026-08-13

**Authors:** Min Xu, Jinling Wu, Min Liu, Xiaoxin Duan, Erhui Sheng, Lina Gu, Lei Liu

**Affiliations:** 1 Affiliated Psychological Hospital of Anhui Medical University, Hefei, China; 2 Hefei Fourth People’s Hospital, Hefei, China

**Keywords:** biological rhythm disruption, chain mediation, emotion regulation difficulties, major depressive disorder, neuroticism, suicidal ideation

## Abstract

**Background:**

Biological rhythm disruption is commonly observed in patients with major depressive disorder (MDD), but the psychological mechanisms linking it to suicidal ideation remain unclear. This study examined whether neuroticism and emotion regulation difficulties mediate this relationship.

**Methods:**

This study included 275 eligible patients with MDD. Biological rhythm disruption, neuroticism, emotion regulation difficulties, and suicidal ideation were assessed using the Biological Rhythms Interview of Assessment in Neuropsychiatry (BRIAN), the NEO Five-Factor Inventory-Neuroticism subscale (NEO-FFI-N), the 16-item Difficulties in Emotion Regulation Scale (DERS-16), and the Self-rating Idea of Suicide Scale (SIOSS), respectively. A chain mediation analysis was conducted using the PROCESS macro (Model 6).

**Results:**

Of the 275 participants, 73.8% reported suicidal ideation. Path analysis showed that biological rhythm disruption had a significant direct association with suicidal ideation (effect = 0.055, 95% CI: 0.007–0.102) and indirect associations through three pathways: (1) via neuroticism alone (effect = 0.086, 95% CI: 0.055–0.121); (2) via emotion regulation difficulties alone (effect = 0.036, 95% CI: 0.017–0.058); and (3) via their serial mediation (effect = 0.021, 95% CI: 0.010–0.034). The total indirect effect accounted for 72.31% of the total effect.

**Conclusions:**

Biological rhythm disruption is not only directly associated with suicidal ideation but also indirectly associated with it through the independent and serial mediating pathways of neuroticism and emotion regulation difficulties in patients with MDD. These findings underscore the potential of interventions that target biological rhythm stabilization, reduce neuroticism-related vulnerability, and enhance emotion regulation to mitigate suicide risk.

## 1 Introduction

Major Depressive Disorder (MDD) is a leading contributor to the global disease burden, characterized by persistent low mood, anhedonia, and cognitive impairment (1, 2). According to the Global Burden of Disease Study 2021, an estimated 332.4 million individuals worldwide were affected by depressive disorders, with an age-standardized prevalence rate of 4006.82 per 100, 000 persons (3). As the leading cause of disability globally (4), MDD is also a major risk factor for suicide. Data indicate that approximately 746, 000 deaths from suicide occurred worldwide in 2021 (5), with over 90% of suicide decedents having at least one mental disorder—among which MDD accounts for the majority of cases (6–9). Suicidal ideation, as the most direct precursor to suicidal behavior, represents a core target for clinical assessment and intervention (10). However, the mechanisms underlying suicidal ideation remain complex and incompletely understood (11, 12). Thus, identifying and elucidating these potentially modifiable mechanisms is of paramount importance for developing precise and effective suicide prevention strategies.

Biological rhythms, regulated by endogenous clocks and external cues such as light, temperature, and social activity, involve periodic changes in physiological and behavioral functions, including sleep-wake cycles, dietary patterns, social activities, hormone secretion, and other important bodily functions regulated by circadian rhythms (13). Disruptions in these rhythms can affect emotions, behaviors, and cognition and may exacerbate mood disorders (14–16). In recent years, biological rhythm disorders have received extensive attention as the core feature and potential pathological mechanism of MDD (17, 18). Indeed, biological rhythm disturbances are highly prevalent among individuals with MDD and are closely linked to both the onset and clinical course of the disorder (19, 20). For instance, sleep-wake cycle alterations are nearly ubiquitous in MDD, with over 90% of patients reporting insomnia or other sleep disruptions (21). Critically, several evidence-based treatments for MDD—such as antidepressant agomelatine (22), bright light therapy (BLT) (23), and interpersonal and social rhythm therapy (IPSRT) (24), exert their therapeutic effects, at least in part, by directly targeting and stabilizing biological rhythms. Therefore, alterations in biological rhythms may represent early, modifiable markers that could improve treatment strategies for MDD.

Notably, beyond its correlation with depressive symptom severity, biological rhythm disruption has been independently associated with increased suicide risk in MDD (25). Early phenomenological studies indicate that specific chronobiological phenotypes confer elevated vulnerability: for example, MDD patients with evening-type tendencies or marked seasonality patterns exhibit significantly higher levels of suicidal ideation compared to their morning-type or non-seasonal counterparts (26). A recent study by Liu et al. (27) demonstrated that patients with MDD exhibited significantly higher levels of dysregulation across all four biological rhythm dimensions (sleep, activity, social, and eating patterns) compared to healthy controls, and the degree of dysregulation was associated with the severity of depression. More importantly, this study found that dysregulation of eating patterns increased the intensity of suicidal ideation in MDD. Converging evidence from a recent multi-center cross-sectional study of 287 patients with mood disorders during depressive episodes further confirms these findings that more severe disruptions in both sleep and eating rhythms were independently associated with suicidal ideation, even after accounting for depressive symptom severity and other clinical factors (28). Collectively, these findings establish biological rhythm disruption as a robust and clinically assessable marker for suicide risk in MDD. However, most existing research has stopped at phenomenological descriptions of this association. A crucial scientific question remains unanswered: Through what psychological mechanism is biological rhythm disruption associated with suicidal ideation? Answering this question is essential for translating these findings into precise intervention targets.

Neuroticism is one of the Big Five personality traits, characterized by emotional instability, negative emotions, and a high sensitivity to stress. It is a key risk factor for the occurrence and recurrence of depression (29, 30). Although neuroticism is traditionally conceptualized as a stable trait, accumulating evidence suggests that its expression is subject to state-dependent fluctuations, particularly during depressive episodes (31, 32). Research has demonstrated a significant association between biological rhythm disruption and neuroticism. Soehner et al. (33) found that individuals with high neuroticism exhibited significantly poorer sleep quality. Tonetti et al. (34) found that evening types showed significantly higher levels of neuroticism than morning types, further supporting the association between biological rhythm types and neuroticism. Furthermore, the relationship between neuroticism and suicidal ideation has been extensively studied. Cameron et al. (35) found that the suicidal ideation group showed a significantly stronger correlation with neuroticism compared to the non-suicidal group, suggesting that neurotic personality may be a key feature distinguishing individuals with suicidal ideation from those without. Meanwhile, the systematic review by Brezo et al. (36) identified neuroticism as an important risk factor for suicidal ideation. These findings provide a theoretical basis for considering neuroticism as a mediator in the relationship between biological rhythm disruption and suicidal ideation. Notably, a longitudinal study by Pawlak et al. (37), which recruited adolescents based on parental history of depression or anxiety disorders, found that after adjusting for age, sex, and baseline symptoms, neuroticism predicted the first onset of depressive disorders but did not predict the occurrence or severity of suicidal ideation. This finding suggests that the effect of neuroticism on suicidal ideation may be indirect, operating through other psychological processes such as emotion regulation difficulties.

Emotion regulation difficulties refer to maladaptive patterns in managing emotional experiences, including a lack of effective regulation strategies, difficulty in impulse control, and insufficient emotional clarity. Emotion regulation ability is critical for maintaining mental health, while emotion regulation difficulties are closely associated with various psychopathological conditions and serve as key proximal risk factors in the formation of suicidal ideation. Patients with depression exhibit persistent emotion regulation impairments (38, 39), characterized by maladaptive strategies such as rumination and expressive suppression, alongside reduced use of adaptive strategies like cognitive reappraisal (40). Emotion dysregulation has been consistently identified as an independent predictor of depression severity (41) and suicide risk (42–44). Neurobiological evidence indicates that disrupted biological rhythms, especially the sleep-wake cycle, may disrupt the neural circuits supporting emotion regulation, such as the prefrontal control of the limbic regions, thereby increasing the susceptibility to emotional dysregulation and impulsive behavior (45, 46), both of which are known suicide risk factors (47, 48).

Furthermore, there is a close theoretical connection between neuroticism and emotional regulation difficulties. Yang et al. (49) conducted an fMRI study and found that individuals with high neuroticism exhibited reduced activity in the dorsomedial prefrontal cortex (dmPFC) and decreased functional connectivity between the amygdala and the dmPFC during a negative emotion regulation task, indicating deficits in cognitive reappraisal ability. These findings provide direct neuroimaging evidence that neuroticism contributes to emotion regulation difficulties. Neuroticism may influence an individual’s cognitive processing styles, thereby affecting their emotional state and suicide risk. Individuals with high neuroticism tend to adopt maladaptive emotion regulation strategies, such as rumination, avoidance behaviors, and emotional suppression. Notably, Vidal-Arenas et al. (50) conducted a cross-national study involving 3, 482 college students from four countries (the United States, Spain, Argentina, and the Netherlands) and found that emotional stability (i.e., low neuroticism) indirectly influences suicidal ideation through the serial mediation of rumination and depressive mood. This suggests the existence of a hierarchical structure in which neuroticism serves as an antecedent of emotion regulation difficulties, and biological rhythm disruption may be associated with heightened neuroticism-related vulnerability, thereby impairing individuals’ emotion regulation ability and ultimately increasing the risk of suicidal ideation.

**Figure 1:**
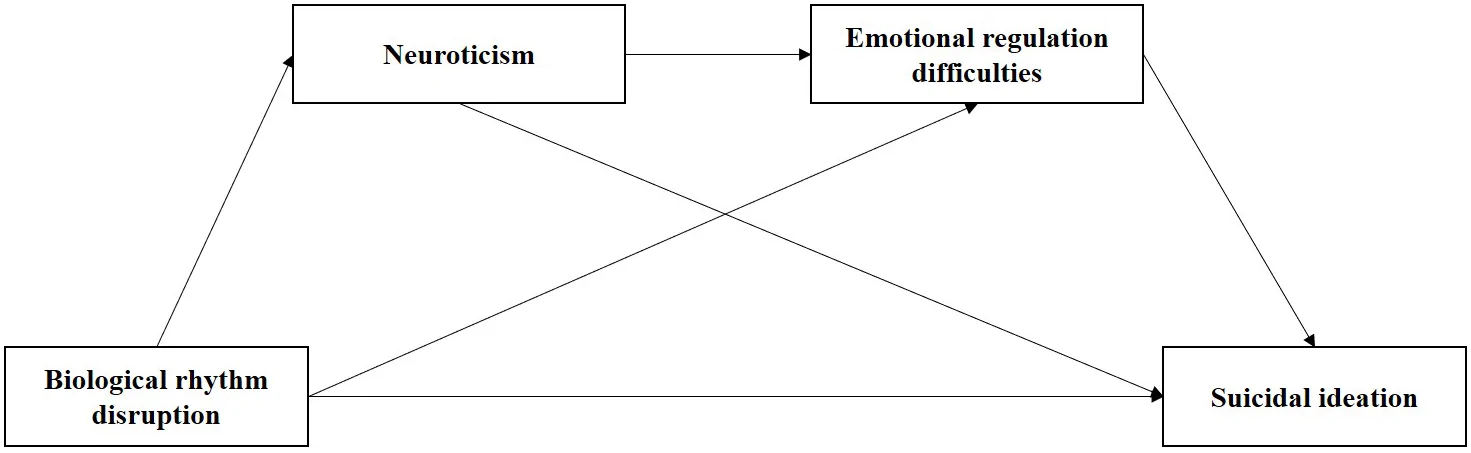
Hypothetical model.

Although previous studies have separately explored the relationships among biological rhythm disruption, neuroticism, emotion regulation difficulties, and suicidal ideation, no study to date has integrated these variables into a comprehensive model to examine how biological rhythm disruption is associated with suicide risk in patients with MDD. Based on the above empirical evidence, we proposed a theoretical hypothesis model (see **Figure 1**) and put forward the following hypotheses: **Hypothesis 1:** Biological rhythm disruption is significantly associated with suicidal ideation (Biological rhythm disruption → suicidal ideation). **Hypothesis 2:** Neuroticism mediate the relationship between biological rhythm disruption and suicidal ideation (biological rhythm disruption → neuroticism → suicidal ideation). **Hypothesis 3:** Emotion regulation difficulties mediate the relationship between biological rhythm disruption and suicidal ideation (biological rhythm disruption → emotion regulation difficulties → suicidal ideation). **Hypothesis 4:** Neuroticism and emotion regulation difficulties jointly function as chain mediators between biological rhythm disruption and suicidal ideation (biological rhythm disruption → neuroticism → emotion regulation difficulties → suicidal ideation).

The present study aims to provide a more comprehensive understanding of the psychological mechanisms linking biological rhythm disruption to suicide risk in MDD. The findings may inform the development of targeted interventions that stabilize biological rhythms and address personality-related vulnerability and emotion regulation deficits, thereby reducing suicide risk in this population.

## 2 Materials and methods

### 2.1 Study design and participants

This cross-sectional study was conducted at the Mental Health Center of a tertiary hospital in China between May 2024 and April 2026. Participants were consecutively recruited from inpatient psychiatric wards. ***Inclusion criteria*** were: (1) clinical diagnosis of MDD according to ICD-10 criteria, confirmed by two psychiatrists with attending physician or higher qualifications; (2) age between 18 and 65 years; (3) a score greater than 20 on the 24-item Hamilton Depression Rating Scale (HAMD-24); and (4) voluntary participation with written informed consent. ***Exclusion criteria*** were: (1) comorbid other types of psychiatric disorders (e.g., bipolar disorder, schizophrenia); (2) intellectual disability; (3) history of alcohol or substance abuse; (4) severe somatic diseases; (5) electroconvulsive therapy in the past 6 months; and (6) engagement in night-shift work.

The study adhered to the principles of the Declaration of Helsinki and was approved by the ethics committee [Approval number: HFSY-IRB-YJ-KYXM-XM (2024-013-001)]. All participants provided written informed consent.

### 2.2 Sample size

Sample size was calculated using G*Power 3.1.9.7 (f² = 0.15, α = 0.05, power = 0.95) based on the number of variables estimated in the full regression model (including sociodemographic and clinical covariates), yielding a minimum requirement of 189 participants. Accounting for a 20% invalid response rate, the target sample size was set at 236. A total of 303 patients were initially enrolled. After excluding 28 incomplete or logically inconsistent questionnaires, 275 valid participants were included (valid response rate: 90.76%), exceeding the required sample size.

### 2.3 Measures

#### 2.3.1 Sociodemographic and clinical characteristics

Sociodemographic information (e.g., gender, age, education level, employment status, marital status) and clinical data (e.g., age of onset, illness duration, number of hospitalizations) were collected using a structured questionnaire developed by the research team based on a review of the literature.

#### 2.3.2 Depressive symptoms

Depressive symptoms were evaluated using the 24-item Hamilton Depression Rating Scale (HAMD-24). The scale comprises 24 items, with higher scores reflecting more severe depressive symptoms. A baseline HAMD-24 total score greater than 20 was used as an inclusion criterion for MDD patients in this study (51). The assessment was performed by trained psychiatrists through a combination of clinical interview and behavioral observation.

#### 2.3.3 Biological rhythm disruption

Biological rhythm disruption was measured by using the Chinese version of the Biological Rhythm Interview of Assessment in Neuropsychiatry (BRIAN) (52). The scale consists of 21 items rated on a 4-point Likert scale (1 = “never” to 4 = “always”), covering four rhythm domains: sleep (items 1–5), activity (items 6–10), social (items 11–14), and eating (items 15–18). Items 19–21 assess predominant biological rhythms and were not included in the total score calculation. Total scores range from 18 to 72, with higher scores indicating greater biological rhythm disruption. The original scale reported a Cronbach’s alpha of 0.898 (52); in this study, the Cronbach’s alpha coefficient was 0.84.

#### 2.3.4 Neuroticism

Neuroticism was measured using the Chinese version of the NEO Five-Factor Inventory-Neuroticism subscale (NEO-FFI-N) (53, 54). The scale consists of 12 items rated on a 5-point Likert scale (1 = “strongly disagree” to 5 = “strongly agree”). Total scores range from 12 to 60, with higher scores indicating higher levels of neuroticism. Archer et al. (55) reported a Cronbach’s alpha of 0.89 for the neuroticism subscale of the NEO-FFI. In this study, Cronbach’s alpha coefficient was 0.78.

#### 2.3.5 Emotion regulation difficulties

Emotion regulation difficulties were assessed using the 16-item version of the Difficulties in Emotion Regulation Scale (DERS-16) (56). This scale consists of 16 items measuring five dimensions: nonacceptance of negative emotions, difficulties engaging in goal-directed behavior, impulse control difficulties, limited access to emotion regulation strategies, and lack of emotional clarity. Items are rated on a 5-point Likert scale (1 = “almost never” to 5 = “almost always”). Total scores range from 16 to 80, with higher scores indicating greater difficulties in emotion regulation. The original scale reported a Cronbach’s alpha of 0.92 (56), in this study, the Cronbach’s alpha coefficient was 0.93.

#### 2.3.6 Suicidal ideation

Suicidal ideation was measured using the Self-rating Idea of Suicide Scale (SIOSS) (57). The scale consists of 26 items grouped into four dimensions: despair, sleep, optimism, and concealment. Items are answered in a “yes” or “no” format. The total score is calculated as the sum of the despair, sleep, and optimism subscales, with a score ≥ 12 indicating the presence of suicidal ideation. Questionnaires with a concealment subscale score greater than 4 were considered invalid and excluded from analysis. The original scale reported a Cronbach’s alpha of 0.79. In this study, the Cronbach’s alpha coefficient for the SIOSS was 0.82.

### 2.4 Data collection procedure

Data were collected using either paper-based questionnaires or electronic devices. All assessments were completed within three days of admission to minimize the confounding effects of psychotropic medication on the study variables.

The data collection procedure consisted of two stages. First, two attending psychiatrists or above confirmed the diagnosis of MDD according to ICD-10 criteria through structured clinical interviews, and assessed depressive symptoms using the 24-item Hamilton Depression Rating Scale (HAMD-24). Second, eligible participants completed a battery of self-report questionnaires administered by trained research assistants following standardized protocols. Participants independently completed the questionnaires in the ward, taking approximately 20–25 minutes to finish all assessments. For participants with limited literacy or physical impairments, research assistants read the questions aloud and provided clarification as needed, without influencing the participants’ responses.

Sociodemographic and clinical information—including age, gender, education level, marital status, employment status, age of onset, illness duration, and number of hospitalizations—was obtained from electronic medical records. All completed questionnaires were reviewed immediately for omissions or inconsistencies to ensure completeness and validity. Data were double-entered and cross-validated to minimize entry errors and ensure accuracy.

### 2.5 Statistical analysis

Statistical analyses were performed using SPSS Version 22.0 (IBM). Harman’s single-factor test was used to assess common method bias. Descriptive statistics were presented as frequencies (percentages) for categorical variables and as mean ± standard deviation (SD) for continuous variables. Normality of the continuous variables was assessed using skewness and kurtosis values, with acceptable normality defined as skewness < |2| and kurtosis < |7| (58). Group comparisons were conducted using independent-samples t-tests or one-way ANOVA, as appropriate. Pearson correlation analyses were performed to examine the bivariate relationships among biological rhythm disruption, neuroticism, emotion regulation difficulties, and suicidal ideation. A chain mediation model was tested using the PROCESS macro (Model 6) for SPSS developed by Hayes (59). The bias-corrected bootstrapping method with 5, 000 resamples was employed to generate 95% confidence intervals (CIs) for the indirect effects. An indirect effect was considered statistically significant if its 95% CI did not include zero. Age, marital status, and age of onset were included as covariates in the model based on the univariate analysis results. To assess the robustness of our findings, we conducted a supplementary mediation analysis adding HAMD-24 total score as a covariate (see **Supplementary Table 1**). Statistical significance was set at a two-tailed p < 0.05.

## 3 Results

### 3.1 Common method bias

Potential common method bias was assessed using Harman’s single-factor test. An unrotated exploratory factor analysis of all items extracted 18 factors with eigenvalues >1, and the first factor accounted for 21.75% of the variance, which is below the 40% criterion, indicating no serious common method bias (60).To further address this issue, we conducted a confirmatory factor analysis (CFA) testing a single-factor model in which all four study variables (BRIAN total, NEO-FFI-N total, DERS total, and SIOSS total) were loaded onto one common factor. The model showed poor fit: χ² = 409.702, df = 2, CFI = 0.000, TLI = 0.000, RMSEA = 0.498, GFI = 0.519, AGFI = 0.199. These fit indices indicate that a single common factor did not account for the observed covariance among the variables, further suggesting that common method bias was unlikely to be a major threat to the validity of our findings.

### 3.2 Sample characteristics

The sample consisted of 275 participants with a mean age of 40.37 years (SD = 13.56; range: 18–65). The majority of participants were female (68.7%) and married (67.3%). The average duration of illness was 6.0 years (SD = 6.51; range: 0.04–30), and the average age of onset was 34.31 years (SD = 13.66; range: 7–63). According to the cutoff score of ≥12 on the SIOSS, 73.8% (203/275) of participants were classified as having suicidal ideation. The SIOSS total score showed acceptable normality (skewness = -0.700, kurtosis = 0.226), supporting the use of parametric analyses. Additional demographic and clinical characteristics are presented in **Table 1**.

**Table 1:** Sample characteristics and univariate analysis of suicidal ideation. (N = 275).

Variables	N (%)	Suicidal ideation	t/F	P
		(mean ± SD)		
Age (years)			5.153	**0.006**
≤30	82 (29.8)	15.13 ± 4.17		
31-45	75 (27.3)	13.93 ± 4.22		
> 45	118 (42.9)	13.19 ± 4.21		
Gender			-1.46	0.145
Male	86 (31.3)	13.42 ± 4.41		
Female	189 (68.7)	14.23 ± 4.19		
Level of education			0.833	0.477
Elementary school or below	57 (20.7)	13.19 ± 4.57		
Junior high school	65 (23.6)	14.17 ± 4.14		
Senior high school	45 (16.4)	14.02 ± 4.67		
College or above	108 (39.3)	14.25 ± 4		
Employment status			1.417	0.158
Unemployed	150 (54.5)	14.31 ± 4.2		
Employed	125 (45.5)	13.58 ± 4.33		
Marital status			2.846	**0.005**
Single	90 (32.7)	15.01 ± 4.14		
Married	185 (67.3)	13.47 ± 4.25		
Residence			-0.061	0.951
Rural	113 (41.1)	13.96 ± 4.25		
Urban	162 (58.9)	13.99 ± 4.29		
Number of children			2.117	0.098
0	83 (30.2)	14.82 ± 4.26		
1	83 (30.2)	13.31 ± 4.59		
2	90 (32.7)	13.66 ± 4.14		
> 2	19 (6.9)	14.68 ± 2.73		
Number of hospitalizations			1.495	0.136
1	204 (74.2)	14.2 ± 3.98		
> 1	71 (25.8)	13.32 ± 4.97		
Age of onset (years)			2.617	**0.009**
≤35	141 (51.3)	14.62 ± 4.1		
>35	134 (48.7)	13.29 ± 4.35		
Duration of illness (years)			-0.687	0.493
≤5	163 (59.3)	13.83 ± 4.65		
>5	112 (40.7)	14.18 ± 3.64		

t/F indicates the results of t-test or one-way analysis of variance. Values in bold are statistically significant.

### 3.3 Univariate analysis of suicidal ideation

As shown in **Table 1**, univariate analyses revealed that age, marital status, and age of onset were significantly associated with suicidal ideation (all p < 0.01). Specifically, younger age (≤30 years), single status, and younger age of onset (≤35 years) were associated with higher levels of suicidal ideation. No significant differences were observed for gender, education level, employment status, residence, number of hospitalizations, or duration of illness (all p > 0.05).

### 3.4 Correlations among variables

Descriptive statistics and the bivariate correlation matrix for all study variables are presented in **Table 2**. Pearson correlation analyses revealed that all variables were significantly correlated with each other (all p < 0.01). Correlation coefficients ranged from 0.467 to 0.64, indicating moderate to strong positive associations among biological rhythm disruption, neuroticism, emotion regulation difficulties, and suicidal ideation. These findings provided preliminary support for the subsequent mediation analyses.

**Table 2:** Description and correlation analysis of key variables.

	Mean ± SD	1	2	3	4
1. BRIAN	46.35 ± 9.64	1			
2. NEO-FFI-N	42.89 ± 7.89	0.478**	1		
3. DERS	47.65 ± 14.85	0.556**	0.602**	1	
4. SIOSS	13.97 ± 4.27	0.467**	0.64**	0.57**	1

**p<0.01. Abbreviations: BRIAN, Biological Rhythm Interview of Assessment in Neuropsychiatry; NEO-FFI-N, NEO Five-Factor Inventory-Neuroticism subscale; DERS, The difficulties in emotion regulation scale; SIOSS, Self-rating Idea of Suicide Scale.

### 3.5 Chain mediation analysis

A chain mediation analysis was conducted using the PROCESS macro (Model 6) developed by Hayes, with 5, 000 bootstrap resamples, to examine the mediating roles of neuroticism and emotion regulation difficulties in the relationship between biological rhythm disruption and suicidal ideation. Biological rhythm disruption was entered as the independent variable, suicidal ideation as the dependent variable, and neuroticism and emotion regulation difficulties as the mediators. Age, marital status, and age of onset were included as covariates.

Regression analysis revealed that biological rhythm disruption positively predicted neuroticism (B = 0.373, p < 0.001), emotion regulation difficulties (B = 0.537, p < 0.001), and suicidal ideation (B = 0.055, p < 0.05). Neuroticism positively predicted emotion regulation difficulties (B = 0.819, p < 0.001) and suicidal ideation (B = 0.229, p < 0.001). Emotion regulation difficulties positively predicted suicidal ideation (B = 0.068, p < 0.001) (see **Table 3**).

**Table 3:** Regression analysis among variables in the chain model.

Regression equation		Fitting index		Significance	
Outcome variable	Predictor variable	R^2^	F	B	t
Neuroticism	Biological rhythm disruption	0.252	22.754***	0.373	8.575***
Emotion regulation difficulties	Biological rhythm disruption	0.456	45.131***	0.537	6.795***
	Neuroticism			0.819	8.369***
Suicidal ideation	Biological rhythm disruption	0.480	41.143***	0.055	2.267*
	Neuroticism			0.229	7.414***
	Emotion regulation difficulties			0.068	3.939***
Suicidal ideation	Biological rhythm disruption	0.242	21.545***	0.197	8.318***

*p<0.05; ***p<0.001, control the age, marital status, and age of onset.

**Figure 2:**
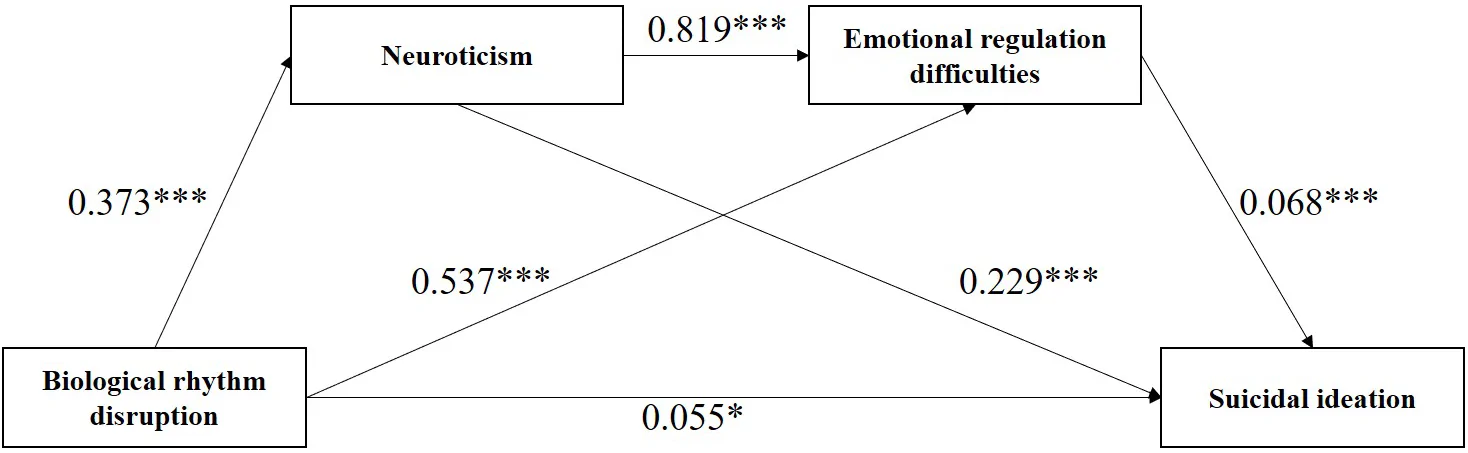
The chain mediating effect of neuroticism and emotional regulation difficulties between biological rhythm disruption and suicidal ideation. Note: *p< 0.05, ***p<0.001.

Bootstrap analysis revealed three significant indirect pathways (see **Table 4**): Indirect effect 1: Biological rhythm disruption → Neuroticism → Suicidal ideation (43.46%); Indirect effect 2: Biological rhythm disruption → Emotion regulation difficulties → Suicidal ideation (18.41%); and Indirect effect 3: Biological rhythm disruption → Neuroticism → Emotion regulation difficulties → Suicidal ideation (10.5%). The total indirect effect accounted for 72.31% of the total effect. The specific path coefficients of the chain mediation model are illustrated in **Figure 2**.

**Table 4:** Bootstrap analysis of significance tests for mediating effects.

Model pathways	Effect	BootSE	BootLLCI	BootULCI	Effect ratio
Total effect	0.197	0.024	0.151	0.244	100.00%
Direct effect	0.055	0.024	0.007	0.102	27.69%
Total indirect effect	0.143	0.019	0.106	0.183	72.31%
Ind1: X → M1 → Y	0.086	0.017	0.055	0.121	43.46%
Ind2: X → M2 → Y	0.036	0.010	0.017	0.058	18.41%
Ind3: X→ M1→ M2→ Y	0.021	0.006	0.010	0.034	10.50%

X, Biological rhythm disruption; M1, Neuroticism; M2, Emotion regulation difficulties; Y, Suicidal ideation. Covariates included age, marital status, and age of onset. Boot SE, Boot LLCI, and Boot ULCI represent the standard error, lower and upper limits of the 95% CI of the indirect effects estimated using the nonparametric percentile Bootstrapped method with bias correction.

## 4 Discussion

To the best of our knowledge, this study is the first to examine the relationships among biological rhythm disruption, neuroticism, emotion regulation difficulties, and suicidal ideation in patients with MDD. Our results showed that biological rhythm disruption was directly associated with suicidal ideation and was also associated with it through three indirect pathways: the independent mediating pathway of neuroticism, the independent mediating pathway of emotion regulation difficulties, and the chain mediating pathway involving both variables. These findings provide a new theoretical perspective for understanding the psychological mechanisms linking biological rhythm disruption to suicide risk in patients with MDD, and offer new directions for future clinical interventions and research.

### 4.1 Prevalence and correlates of suicidal ideation

In the present study, 73.8% of patients with MDD reported suicidal ideation, which is slightly higher than the 66.02% reported by Zhou et al. (61) in college students with MDD. A possible explanation is that our sample consisted of hospitalized patients with more severe illness. Additionally, differences in measurement instruments may have contributed to this discrepancy.

We found that younger age was associated with higher levels of suicidal ideation. This finding is highly consistent with the results of Liu et al. (62), who studied 151 first-episode, drug-naïve patients with MDD and reported that age was a significant predictor of suicidal ideation, with younger age associated with higher suicide risk. Similarly, the scoping review by Su et al. (10) identified younger age as a consistent risk factor for suicidal ideation or behavior among Chinese patients with MDD. Potential explanations include that younger patients may face greater academic, occupational, and interpersonal pressures; have less mature illness cognition and coping abilities; and may be more susceptible to negative information. We also found that being single was associated with higher levels of suicidal ideation. Liu et al. (62) similarly reported that being single was an important predictor in their predictive model of suicidal ideation. Marital relationships may provide emotional support, economic assistance, and social integration, thereby buffering the impact of stress on mental health. Single individuals may lack adequate coping resources and emotional support during depressive episodes, which may explain their higher levels of suicidal ideation. Furthermore, we found that early onset of MDD (≤35 years) was associated with higher levels of suicidal ideation. This finding is consistent with the results of Olgiati et al. (63), who reported that patients with severe suicidal ideation had an earlier age of onset. Early-onset depression may be associated with longer illness duration, more frequent relapses, and poorer overall prognosis, all of which may contribute to increased suicide risk.

### 4.2 Effect of biological rhythm disruption on suicidal ideation

We found that even after controlling for age, marital status, and age of onset, biological rhythm disruption remained significantly associated with suicidal ideation. This indicates that greater biological rhythm disruption is associated with more severe suicidal ideation in patients with MDD, independent of demographic and clinical covariates. This finding is consistent with previous studies (27, 28) and further confirms that biological rhythm disruption is an independent risk factor for suicidal ideation in MDD patients.

Notably, the direct effect of biological rhythm disruption on suicidal ideation (β = 0.055, p < 0.05) remained significant even after accounting for the mediating roles of neuroticism and emotion regulation difficulties, suggesting that biological rhythm disruption may also be linked to suicidal ideation through pathways not captured by our model. The direct effect accounted for 27.69% of the total effect, indicating that while psychological mechanisms play a predominant role, there is a substantial direct component that warrants further investigation. From a clinical perspective, this finding implies that interventions targeting biological rhythm stabilization, such as interpersonal and social rhythm therapy or bright light therapy, may have direct protective effects against suicidal ideation, regardless of their impact on personality or emotion regulation.

### 4.3 Mediating role of neuroticism

Mediation analysis revealed that neuroticism mediated the relationship between biological rhythm disruption and suicidal ideation, confirming our hypothesis. Biological rhythm disruption may be associated with heightened neuroticism-related vulnerability by impairing emotional stability and increasing stress sensitivity, which in turn enhances the intensity of suicidal ideation.

The significant association between neuroticism and suicidal ideation observed in this study is consistent with the systematic review by Brezo et al. (36), further confirming neuroticism as a core personality risk factor for suicide. More importantly, the longitudinal study by Gorgol et al. (64) found that neuroticism moderated the relationship between seasonal changes in circadian rhythm and depressive symptoms. Together, these findings converge on the central role of neuroticism in connecting biological rhythms to mental health outcomes. However, the longitudinal study by Pawlak et al. (37), which recruited adolescents based on parental history of depression or anxiety disorders, found that after adjusting for baseline symptoms, neuroticism did not predict the occurrence of suicidal ideation. This discrepancy may be attributed to differences in sample characteristics: Pawlak et al. studied adolescents at familial risk for mood disorders who had not yet met diagnostic criteria for MDD, whereas the present study focused on clinically diagnosed MDD patients. In a population that has already developed clinical depression, neuroticism-related vulnerability may show a stronger association with suicidal ideation. Therefore, we recommend that clinical practice attend to the vulnerability of neuroticism in depressed patients. Through targeted psychological interventions such as cognitive-behavioral therapy, it may be possible to reduce neuroticism levels and thereby decrease suicide risk.

### 4.4 Mediating role of emotion regulation difficulties

We also found that emotion regulation difficulties played a significant mediating role between biological rhythm disruption and suicidal ideation, supporting the important role of emotion regulation difficulties as a proximal risk factor in the formation of suicidal ideation. Consistent with our findings, Palagini et al. (65) found in patients with bipolar disorder that circadian rhythm disruption indirectly associated with depressive symptoms and suicide risk by impairing resilience and increasing emotion regulation difficulties, particularly impulsivity, providing transdiagnostic empirical support for this pathway. The experimental sleep deprivation study by Simon et al. (66) revealed the neural mechanism underlying this process: sleep restriction impairs prefrontal regulation of the amygdala and lowers the threshold for emotional activation, thereby contributing to decreased emotion regulation capacity. The cognitive model of emotion regulation suggests that maladaptive emotion regulation strategies are key cognitive risk factors for suicide (67). Individuals who cannot effectively regulate their emotions when faced with biological rhythm disruption are more likely to develop suicidal thoughts.

### 4.5 Chain mediating role of neuroticism and emotion regulation difficulties

Our results showed that neuroticism and emotion regulation difficulties played a chain mediating role between biological rhythm disruption and suicidal ideation. From a theoretical hierarchy perspective, neuroticism-related vulnerability, as a state-dependent expression of the personality trait, reflects an individual’s susceptibility to negative emotions during depressive episodes, whereas difficulties in emotion regulation represent an acquired cognitive-behavioral pattern closer to clinical outcomes. In this study, neuroticism significantly associated with emotion regulation difficulties (β = 0.819, p < 0.001), a finding consistent with the neuroimaging study by Yang et al. (49), which demonstrated that individuals with high neuroticism exhibited reduced dmPFC activity and decreased amygdala-dmPFC connectivity during a negative emotion regulation task, suggesting that neuroticism may impair prefrontal regulation of the amygdala, thereby leading to decreased emotion regulation capacity. The cross-national study by Vidal-Arenas et al. (50) found that emotional stability (i.e., low neuroticism) indirectly influenced suicidal ideation through the chain mediation of rumination and depressed mood. Building on these findings, our study extends the literature by using biological rhythm disruption as a more upstream starting variable and, for the first time in MDD patients, reveals a cascading pathway from biological rhythm disruption to personality-related vulnerability, to emotion regulation processes, and finally to suicidal ideation. The existence of this chain mediating pathway highlights the potential value of comprehensive, multi-target interventions that simultaneously address biological rhythms, personality-related vulnerability, and emotion regulation.

### 4.6 Limitations and future directions

Several limitations of this study should be considered when interpreting the results. First, although our chain mediation model was constructed based on theoretical assumptions, the cross-sectional design cannot confirm causal directions. We acknowledge that the proposed ordering of variables remains theoretical and that alternative temporal sequences cannot be ruled out. Future longitudinal studies are warranted to establish the directionality of these associations. Second, the sample was drawn from a single center and consisted entirely of Chinese Han individuals. Generalization of the findings to other populations (e.g., other ethnicities, different cultural backgrounds) should be made with caution. Future studies should validate our findings in multi-center, cross-cultural samples. Third, all variables were measured using self-report scales, which may be subject to recall bias. In particular, although the BRIAN scale has good reliability and validity, self-reported biological rhythm disruption may differ from objective measurements. Future studies should combine objective physiological indicators (e.g., actigraphy, cortisol rhythms, core body temperature). Fourth, psychotropic medication status was not included as a covariate because data were collected within three days of admission, before medication regimens had stabilized. Nevertheless, we acknowledge that medication use represents a non-negligible confounding factor. Future research should incorporate objective measures of medication use to further address this limitation. Finally, other potential mediating or moderating variables were not considered. Our mediation model explained 72.31% of the total effect, leaving approximately 30% of the effect unexplained. Future research should explore whether other potential variables (e.g., social support, resilience, childhood trauma, inflammatory markers) may also play mediating or moderating roles in the relationship between biological rhythm disruption and suicidal ideation.

## 5 Conclusion

In summary, this study provides new evidence that neuroticism and emotion regulation difficulties serve as both independent and chain mediators associated with the relationship between biological rhythm disruption and suicidal ideation in patients with MDD. These findings offer a new theoretical perspective for understanding the psychological mechanisms associated with biological rhythm disruption and suicide risk in MDD, and provide empirical support for the development of targeted intervention strategies. Future research should employ longitudinal designs and objective measurement methods to further validate our findings and explore the generalizability of the model across cultures.

## Data Availability

The original contributions presented in the study are included in the article/**Supplementary Material**. Further inquiries can be directed to the corresponding authors.

## References

[1] Marx W Penninx B Solmi M Furukawa TA Firth J Carvalho AF . Major depressive disorder. Nat Rev Dis Primers. (2023) 9:44. doi: 10.1038/s41572-023-00454-1 37620370

[2] Wang L Li L Zhang R Zhao J Wei M Nie Z . Comparison of disease burden of major depressive disorder in China and globally from 1991 to 2021. J Affect Disord. (2025) 390:119816. doi: 10.1016/j.jad.2025.119816 40617487

[3] Rong J Wang X Cheng P Li D Zhao D . Global, regional and national burden of depressive disorders and attributable risk factors, from 1990 to 2021: results from the 2021 Global Burden of Disease study. Br J Psychiatry. (2025) 227:688–97. doi: 10.1192/bjp.2024.266 39809717

[4] Friedrich MJ . Depression is the leading cause of disability around the world. JAMA. (2017) 317:1517. doi: 10.1001/jama.2017.3826 28418490

[5] Davis Weaver N Bertolacci GJ Rosenblad E Ghoba S Cunningham M Ikuta KS . Global, regional, and national burden of suicide, 1990–2021: a systematic analysis for the Global Burden of Disease Study 2021. Lancet Public Health. (2025) 10:e189–202. doi: 10.1016/S2468-2667(25)00006-4 39986290 PMC11876099

[6] Heuschen C Mocking RJT Zantvoord JB Figueroa CA Schene AH Denys D . Suicidal ideation in remitted major depressive disorder predicts recurrence. J Psychiatr Res. (2022) 151:65–72. doi: 10.1016/j.jpsychires.2022.04.005 35461004

[7] Bertolote JM Fleischmann A . Suicide and psychiatric diagnosis: a worldwide perspective. World Psychiatry. (2002) 1:181–5. PMC1489848 16946849

[8] Arsenault-Lapierre G Kim C Turecki G . Psychiatric diagnoses in 3275 suicides: a meta-analysis. BMC Psychiatry. (2004) 4:37. doi: 10.1186/1471-244x-4-37 15527502 PMC534107

[9] Cavanagh JT Carson AJ Sharpe M Lawrie SM . Psychological autopsy studies of suicide: a systematic review. Psychol Med. (2003) 33:395–405. doi: 10.1017/s0033291702006943 12701661

[10] Su Y Ye C Xin Q Si T . Major depressive disorder with suicidal ideation or behavior in Chinese population: A scoping review of current evidence on disease assessment, burden, treatment and risk factors. J Affect Disord. (2023) 340:732–42. doi: 10.1016/j.jad.2023.08.106 37619652

[11] Kang N You J Huang J Ren Y Lin MP Xu S . Understanding the pathways from depression to suicidal risk from the perspective of the interpersonal-psychological theory of suicide. Suicide Life Threat Behav. (2019) 49:684–94. doi: 10.1111/sltb.12455 29578277

[12] Zortea TC Cleare S Melson AJ Wetherall K O'Connor RC . Understanding and managing suicide risk. Br Med Bull. (2020) 134:73–84. doi: 10.1093/bmb/ldaa013 32435794

[13] Salvatore P Indic P Murray G Baldessarini RJ . Biological rhythms and mood disorders. Dialogues Clin Neurosci. (2012) 14:369–79. doi: 10.31887/DCNS.2012.14.4/psalvatore 23393414 PMC3553575

[14] Crouse JJ Carpenter JS Song YJC Hockey SJ Naismith SL Grunstein RR . Circadian rhythm sleep-wake disturbances and depression in young people: implications for prevention and early intervention. Lancet Psychiatry. (2021) 8:813–23. doi: 10.1016/s2215-0366(21)00034-1 34419186

[15] Sabet SM Dautovich ND Dzierzewski JM . The rhythm is gonna get you: Social rhythms, sleep, depressive, and anxiety symptoms. J Affect Disord. (2021) 286:197–203. doi: 10.1016/j.jad.2021.02.061 33735764 PMC8058264

[16] Koning E Vorstman J McIntyre RS Brietzke E . Characterizing eating behavioral phenotypes in mood disorders: a narrative review. Psychol Med. (2022) 52:2885–98. doi: 10.1017/s0033291722002446 36004528 PMC9693712

[17] Manosso LM Duarte LA Martinello NS Mathia GB Réus GZ . Circadian rhythms and sleep disorders associated to major depressive disorder: Pathophysiology and therapeutic opportunities. CNS Neurol Disord Drug Targets. (2024) 23:1085–100. doi: 10.2174/0118715273254093231020052002 37885113

[18] Smith WK Zhong ZM Wang WT Hassan NU Khan M Wang H . Circadian rhythms and their roles in the pathogenesis and treatment of depression. Sheng Li Xue Bao. (2025) 77:689–711. doi: 10.13294/j.aps.2025.0067 40855800

[19] Dollish HK Tsyglakova M McClung CA . Circadian rhythms and mood disorders: Time to see the light. Neuron. (2024) 112:25–40. doi: 10.1016/j.neuron.2023.09.023 37858331 PMC10842077

[20] Tonon AC Constantino DB Amando GR Abreu AC Francisco AP de Oliveira MAB . Sleep disturbances, circadian activity, and nocturnal light exposure characterize high risk for and current depression in adolescence. Sleep. (2022) 45:zsac104. doi: 10.1093/sleep/zsac104 35522984

[21] Geoffroy PA Palagini L . Biological rhythms and chronotherapeutics in depression. Prog Neuro-Psychopharmacol Biol Psychiatry. (2021) 106:110158. doi: 10.1016/j.pnpbp.2020.110158 33152388

[22] Guo P Xu Y Lv L Feng M Fang Y Huang WQ . A multicenter, randomized controlled study on the efficacy of agomelatine in ameliorating anhedonia, reduced motivation, and circadian rhythm disruptions in patients with major depressive disorder (MDD). Ann Gen Psychiatry. (2023) 22:46. doi: 10.1186/s12991-023-00473-y 37957751 PMC10642047

[23] Kosanovic Rajacic B Sagud M Pivac N Begic D . Illuminating the way: the role of bright light therapy in the treatment of depression. Expert Rev Neurother. (2023) 23:1157–71. doi: 10.1080/14737175.2023.2273396 37882458

[24] Crowe M Inder M Douglas K Carlyle D Wells H Jordan J . Interpersonal and social rhythm therapy for patients with major depressive disorder. Am J Psychother. (2020) 73:29–34. doi: 10.1176/appi.psychotherapy.20190024 31752508

[25] Walsh RFL Maddox MA Smith LT Liu RT Alloy LB . Social and circadian rhythm dysregulation and suicide: A systematic review and meta-analysis. Neurosci Biobehav Rev. (2024) 158:105560. doi: 10.1016/j.neubiorev.2024.105560 38272337 PMC10982958

[26] Bahk YC Han E Lee SH . Biological rhythm differences and suicidal ideation in patients with major depressive disorder. J Affect Disord. (2014) 168:294–7. doi: 10.1016/j.jad.2014.07.001 25080393

[27] Liu D Zhang M Ding L Huang J Wang Y Su Y . Relationship between biological rhythm dysregulation and suicidal ideation in patients with major depressive disorder. BMC Psychiatry. (2024) 24:87. doi: 10.1186/s12888-024-05528-2 38297264 PMC10832079

[28] Yu J Shan X Du L . Association between biological rhythm disturbances and suicidal ideation in mood disorders: A cross-sectional study in China. Sci Rep. (2025) 15:37275. doi: 10.1038/s41598-025-21303-z 41136547 PMC12552720

[29] Altaweel N Upthegrove R Surtees A Durdurak B Marwaha S . Personality traits as risk factors for relapse or recurrence in major depression: a systematic review. Front Psychiatry. (2023) 14:1176355. doi: 10.3389/fpsyt.2023.1176355 37215669 PMC10196019

[30] Kotov R Gamez W Schmidt F Watson D . Linking "big" personality traits to anxiety, depressive, and substance use disorders: a meta-analysis. Psychol Bull. (2010) 136:768–821. doi: 10.1037/a0020327 20804236

[31] Karsten J Penninx BWJH Riese H Ormel J Nolen WA Hartman CA . The state effect of depressive and anxiety disorders on big five personality traits. J Psychiatr Res. (2012) 46:644–50. doi: 10.1016/j.jpsychires.2012.01.024 22349302

[32] Costa PT Bagby RM Herbst JH McCrae RR . Personality self-reports are concurrently reliable and valid during acute depressive episodes. J Affect Disord. (2005) 89:45–55. doi: 10.1016/j.jad.2005.06.010 16203041

[33] Soehner AM Kennedy KS Monk TH . Personality correlates with sleep-wake variables. Chronobiol Int. (2007) 24:889–903. doi: 10.1080/07420520701648317 17994344

[34] Tonetti L Fabbri M Natale V . Relationship between circadian typology and big five personality domains. Chronobiol Int. (2009) 26:337–47. doi: 10.1080/07420520902750995 19212845

[35] Cameron S Brown VJ Dritschel B Power K Cook M . Understanding the relationship between suicidality, current depressed mood, personality, and cognitive factors. Psychol Psychother. (2017) 90:530–49. doi: 10.1111/papt.12123 28296207

[36] Brezo J Paris J Turecki G . Personality traits as correlates of suicidal ideation, suicide attempts, and suicide completions: a systematic review. Acta Psychiatr Scand. (2006) 113:180–206. doi: 10.1111/j.1600-0447.2005.00702.x 16466403

[37] Pawlak M Schmidtler H Kopala-Sibley DC . Neuroticism and extraversion as predictors of first-lifetime onsets of depression, anxiety, and suicidality in high-risk adolescents. Dev Psychopathol. (2025) 37:529–40. doi: 10.1017/s0954579424000130 38351640

[38] McRae K Gross JJ . Emotion regulation. Emotion. (2020) 20:1–9. doi: 10.1037/emo0000703 31961170

[39] Liu DY Thompson RJ . Selection and implementation of emotion regulation strategies in major depressive disorder: An integrative review. Clin Psychol Rev. (2017) 57:183–94. doi: 10.1016/j.cpr.2017.07.004 28739277

[40] Song X Zhang XY Du F . Emotion regulation repertoires predict the risk of major depressive disorder. J Affect Disord. (2025) 376:251–9. doi: 10.1016/j.jad.2025.01.126 39892756

[41] Snyder MD Zimmerman M . The contributions of social functioning and emotion dysregulation to depression severity in patients with depressive disorders. J Affect Disord. (2026) 392:120191. doi: 10.1016/j.jad.2025.120191 40914536

[42] Rania M Monell E Sjölander A Bulik CM . Emotion dysregulation and suicidality in eating disorders. Int J Eat Disord. (2021) 54:313–25. doi: 10.1002/eat.23410 33205495 PMC7984062

[43] Rogante E Cifrodelli M Sarubbi S Costanza A Erbuto D Berardelli I . The role of emotion dysregulation in understanding suicide risk: A systematic review of the literature. Healthcare (Basel). (2024) 12:169. doi: 10.3390/healthcare12020169 38255058 PMC10815449

[44] Phalen P Kimhy D Jobes D Bennett M . Emotional distress and dysregulation as treatment targets to reduce suicide in psychosis: a scoping review. Eur Arch Psychiatry Clin Neurosci. (2024) 274:955–61. doi: 10.1007/s00406-023-01675-x 37597022 PMC12086700

[45] Gruber R Cassoff J . The interplay between sleep and emotion regulation: conceptual framework empirical evidence and future directions. Curr Psychiatry Rep. (2014) 16:500. doi: 10.1007/s11920-014-0500-x 25200984

[46] Francis TC Porcu A . Emotionally clocked out: cell-type specific regulation of mood and anxiety by the circadian clock system in the brain. Front Mol Neurosci. (2023) 16:1188184. doi: 10.3389/fnmol.2023.1188184 37441675 PMC10333695

[47] Kulacaoglu F Izci F . The effect of emotional dysregulation and impulsivity on suicidality in patients with bipolar disorder. Psychiatr Danub. (2022) 34:706–14. doi: 10.24869/psyd.2022.706 36548885

[48] Gao Y Liu J Liu X Wang Y Qiu S . Dimensions of family stress and repetitive nonsuicidal self-injury in adolescence: Examining the interactive effects of impulsivity and emotion dysregulation. Child Abuse Negl. (2024) 152:106804. doi: 10.1016/j.chiabu.2024.106804 38636157

[49] Yang J Mao Y Niu Y Wei D Wang X Qiu J . Individual differences in neuroticism personality trait in emotion regulation. J Affect Disord. (2020) 265:468–74. doi: 10.1016/j.jad.2020.01.086 32090774

[50] Vidal-Arenas V Bravo AJ Ortet-Walker J Ortet G Mezquita L Ibáñez MI . Neuroticism, rumination, depression and suicidal ideation: A moderated serial mediation model across four countries. Int J Clin Health Psychol. (2022) 22:100325. doi: 10.1016/j.ijchp.2022.100325 35950010 PMC9343412

[51] Chen Y Jiang ZY Dong GZ Zhang WY Wang K Yang HY . Using machine learning and the HAMD-24 scale to predict suicide ideation in depressed patients. Psychol Res Behav Manag. (2025) 18:2153–65. doi: 10.2147/prbm.S537582 41112937 PMC12531378

[52] He S Ding L He K Zheng B Liu D Zhang M . Reliability and validity of the Chinese version of the biological rhythms interview of assessment in neuropsychiatry in patients with major depressive disorder. BMC Psychiatry. (2022) 22:834. doi: 10.1186/s12888-022-04487-w 36581864 PMC9798705

[53] Costa PT Jr. McCrae RR . Domains and facets: hierarchical personality assessment using the revised NEO personality inventory. J Pers Assess. (1995) 64:21–50. doi: 10.1207/s15327752jpa6401_2 16367732

[54] Costa PT Mccrae RR . Revised NEO personality inventory (NEO PI-R) and NEP five-factor inventory (NEO-FFI): professional manual. Odessa, FL: Psychological Assessment Resources (1992).

[55] Archer N Brown RG Boothby H Foy C Nicholas H Lovestone S . The NEO-FFI is a reliable measure of premorbid personality in patients with probable Alzheimer's disease. Int J Geriatr Psychiatry. (2006) 21:477–84. doi: 10.1002/gps.1499 16676294

[56] Bjureberg J Ljótsson B Tull MT Hedman E Sahlin H Lundh LG . Development and validation of a brief version of the Difficulties in Emotion Regulation Scale: The DERS-16. J Psychopathol Behav Assess. (2016) 38:284–96. doi: 10.1007/s10862-015-9514-x 27239096 PMC4882111

[57] Xia CY Wang DB Wu SQ Ye JH . Preliminary formulation of the suicide ideation self-rating scale [in Chinese. J Clin Psychiatry. (2002) 12:100–2.

[58] Curran PJ West SG Finch JF . The robustness of test statistics to nonnormality and specification error in confirmatory factor analysis. Psychol Methods. (1996) 1:16–29. doi: 10.1037/1082-989x.1.1.16 27371692

[59] Hayes AF . An index and test of linear moderated mediation. Multivariate Behav Res. (2015) 50:1–22. doi: 10.1080/00273171.2014.962683 26609740

[60] Podsakoff PM MacKenzie SB Podsakoff NP . Sources of method bias in social science research and recommendations on how to control it. Annu Rev Psychol. (2012) 63:539–69. doi: 10.1146/annurev-psych-120710-100452 21838546

[61] Zhou SC Luo D Wang XQ Zhu J Wu S Sun T . Suicidal ideation in college students having major depressive disorder: Role of childhood trauma, personality and dysfunctional attitudes. J Affect Disord. (2022) 311:311–8. doi: 10.1016/j.jad.2022.05.085 35597473

[62] Liu X Liu Y Gao Y Zhang C Zhou C Li S . Factors associated with suicidal ideation in drug-naïve patients with major depressive disorder. J Affect Disord. (2025) 380:802–7. doi: 10.1016/j.jad.2025.03.111 40120956

[63] Olgiati P Fanelli G Serretti A . Clinical correlates and prognostic implications of severe suicidal ideation in major depressive disorder. Int Clin Psychopharmacol. (2023) 38:201–8. doi: 10.1097/yic.0000000000000461 36853754 PMC10234328

[64] Gorgol J Stolarski M Jankowski KS . The moderating role of personality traits in the associations between seasonal fluctuations in chronotype and depressive symptoms. Chronobiol Int. (2022) 39:1078–86. doi: 10.1080/07420528.2022.2067000 35450500

[65] Palagini L Miniati M Marazziti D Massa L Grassi L Geoffroy PA . Circadian rhythm alterations may be related to impaired resilience, emotional dysregulation and to the severity of mood features in bipolar I and II disorders. Clin Neuropsychiatry. (2022) 19:174–86. doi: 10.36131/cnfioritieditore20220306 35821870 PMC9263680

[66] Simon EB Oren N Sharon H Kirschner A Goldway N Okon-Singer H . Losing neutrality: The neural basis of impaired emotional control without sleep. J Neurosci. (2015) 35:13194–205. doi: 10.1523/jneurosci.1314-15.2015 26400948 PMC6605430

[67] Hasking P Whitlock J Voon D Rose A . A cognitive-emotional model of NSSI: using emotion regulation and cognitive processes to explain why people self-injure. Cognit Emot. (2017) 31:1543–56. doi: 10.1080/02699931.2016.1241219 27702245

