# Supplementary material for: Association between biological rhythm disruption and suicidal ideation in patients with major depressive disorder: a chain mediation of neuroticism and emotion regulation difficulties
Source: Front Psychiatry. 2026 Aug 13;17:1895657. doi: 10.3389/fpsyt.2026.1895657 (PMC13519599; doi:10.3389/fpsyt.2026.1895657)
Supplement: Supplementary file 1 [file Table1.docx]

**Table S1. Bootstrap analysis of significance tests for mediating effects.**

| Model pathways | Effect | BootSE | BootLLCI | BootULCI | Effect ratio |
| --- | --- | --- | --- | --- | --- |
| Total effect | 0.186 | 0.024 | 0.138 | 0.233 | 100.00% |
| Direct effect | 0.048 | 0.024 | 0.001 | 0.096 | 25.81% |
| Total indirect effect | 0.138 | 0.019 | 0.101 | 0.177 | 74.19% |
| Ind1: X → M1 → Y | 0.086 | 0.017 | 0.056 | 0.122 | 46.24% |
| Ind2: X → M2 → Y | 0.033 | 0.01 | 0.015 | 0.053 | 17.74% |
| Ind3: X→ M1→ M2→ Y | 0.019 | 0.006 | 0.009 | 0.032 | 10.22% |

**Note:** X = Biological rhythm disruption, M1 = Neuroticism, M2 = Emotion regulation difficulties, Y = Suicidal ideation. Covariates included **age, marital status, age of onset, and HAMD-24 total score**. Boot SE, Boot LLCI, and Boot ULCI represent the standard error, lower, and upper limits of the 95 % CI of the indirect effects estimated using the nonparametric percentile Bootstrapped method with bias correction.
